# The effects of feeding pelleted timothy hay to ameliorate the duration, frequency, and latency to start cross-sucking behavior in paired dairy calves

**DOI:** 10.3168/jdsc.2025-0789

**Published:** 2025-10-03

**Authors:** G.D. Plaugher, M.C. Cantor

**Affiliations:** Department of Animal Science, The Pennsylvania State University, University Park, PA 16803

## Abstract

Cross-sucking is an abnormal oral behavior that is a potential barrier to the adoption of social housing of calves. The objective of this randomized control trial was to assess whether providing pelleted timothy hay to pair-housed dairy calves could affect the duration, frequency, and latency to start cross-sucking behavior surrounding the daily milk feedings for the first 21 d after pairing. We enrolled 24 calves (n = 12 pairs; 6 pairs negative control [CON], 6 pairs timothy hay pellets [Hay]) at pairing at 5 ± 3 d of age. Calves were fed 7.4 L/d of milk replacer by bottle in 2 equal feedings and access to water by bucket from birth and had access to calf starter grain by bucket and by bottle ad libitum after pairing. Orts were recorded daily by scale for hay and calf starter intake. Calves were video recorded for 21 consecutive days after pairing. Two trained observers (ĸ = 0.80) watched cross-sucking events twice daily for the hour before and 2 h after each milk feeding for cross-sucking behavior using BORIS software. A cross-sucking bout was defined as one calf suckling on the navel, udder area, or between the back legs of the receiving calf and ended when the cross-sucker broke contact with the defined areas of the receiving calf for ≥10 s. The cross-sucking bouts were summarized into the daily frequency for each pair, and the duration of the bouts was summed into the daily duration by pair. The latency to first cross-suck was the first day on which a cross-sucking bout was observed within a pair. Data were non-normally distributed (Shapiro–Wilk criteria <0.95) and were common log-transformed with a 1.0 correction factor for model fit. We used mixed linear regression models to evaluate the association of Hay with the cross-sucking duration, cross-sucking frequency, day, and the Hay × day interaction, repeating by day, and using pair as the subject. Latency to begin cross-sucking was assessed using Pearson's chi-squared test. The back-transformed geometric means minus the correction factor and 95% CI of the back-transformed data are reported. We observed that Hay and the Hay × day interaction were not associated with the duration or frequency of daily cross-sucking bouts surrounding milk feeding. The geometric mean daily duration of cross-sucking surrounding milk feeding for CON was 54.7 (95% CI: 49.3 to 60.5 s) and for Hay was 59.4 (95% CI: 53.3 to 65.5 s). The geometric mean daily frequency of cross-sucking bouts surrounding milk feeding for CON was 7.8 (95% CI: 7.5 to 8.1 bouts) and for Hay was 8.1 (95% CI: 7.7 to 8.4 bouts). The latency to cross-suck was not associated with the provision of the Hay pellets and occurred by a maximum of 2 d after pairing (95% CI: 0.81 to 2.52 d). However, the average pelleted hay DMI throughout the study was negligible (Hay 89.41 ± 99.89 g/d; mean ± SD), reaching 220.51 ± 108.81 g/d on d 21. Our results suggest that offering timothy hay to pair-housed calves was not associated with cross-sucking behavior, though the behavior occurred for a short duration surrounding milk feeding, and was variable by pair.

## Highlights

• We investigated whether timothy hay ameliorates cross-sucking in paired calves (n = 24).
• Hay was not associated with bout duration, frequency, or latency to cross-suckle.
• All paired calves cross-suckled within 0 to 1 days after pairing.

## Graphical Abstract

**Figure:**
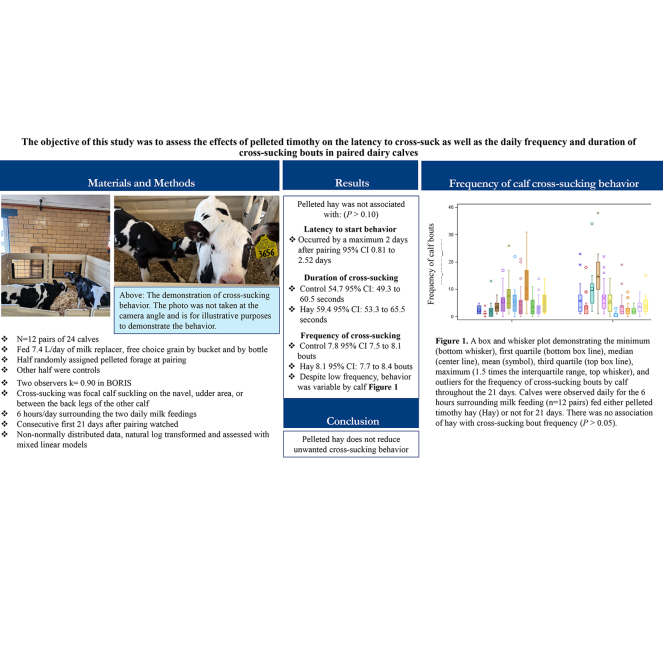
**Summary:** We investigated the effects of providing paired calves with a pelleted hay or a negative control on the duration, frequency, and latency to begin cross-sucking (i.e., non-nutritive suckling of another calf) surrounding milk feedings. We enrolled calves at pairing at 5 days of age (n = 12 pairs; 6 pairs fed pelleted timothy hay, 6 pairs fed none) and followed their cross-sucking behavior for the hour before and the 2 hours after milk feeding (6 hours/day) for the first 21 days. We found no association of pelleted timothy hay with duration, frequency, or latency to begin cross-sucking, but cross-sucking was variable by pair. We suggest that pelleted timothy hay is not useful for reducing unwanted cross-sucking behavior in pair-housed calves.

Group housing of dairy calves is important for their cognitive and social development (as reviewed by Costa et al., 2016), yet non-nutritive oral behaviors such as cross-sucking can develop in these housing systems. Cross-sucking has been defined as when a calf suckles on the underside of another calf (de Passillé et al., 2011), and this behavior has been associated with poor calf health and comfort by producers (Doyle et al., 2024). This behavior may result in injury to calves and early removal from the herd (Mahmoud et al., 2016), which is undesirable for producers. Further research on strategies to reduce this behavior is necessary to provide optimal calf welfare in social housing scenarios.

One tactic that has been previously explored is providing socially housed calves with forage to attempt to ameliorate cross-sucking bouts. It is hypothesized that hay may reduce cross-sucking in socially housed calves due to increased rumination, chewing time, or gut fill (Horvath and Miller-Cushon, 2019; Ude et al., 2011; Zhang et al., 2021). The previous studies investigated the provision of long-stem hay to calves, though other physical forms of hay are available. We chose pellets because Arave et al. (1983) found that pellets are a preferred feedstuff in heifers over ground feed, and Molaei et al. (2021) observed that pelleted hay does not negatively affect rumen fermentation in calves compared with chopped hay. Plus, young calves that do not have hay provided eat their bedding (Wang et al., 2022), suggesting a desire for fiber. Perhaps offering a timothy hay pellet when there are higher levels of fiber in pelleted timothy hay than alfalfa hay (NASEM, 2021) may re-direct calves seeking oral stimulation. Research investigating pelleted timothy hay as a tool to reduce cross-sucking is warranted because some farmers do not have long-stem hay available to feed their calves.

Cross-sucking is a barrier to producers who consider social housing for dairy calves (Doyle et al., 2024). Pelleted hay may serve as a suitable tactic to reduce cross-sucking behavior without affecting growth performance or calf health (Kincaid, 1980; Jahani-Moghadam et al., 2015; Molaei et al., 2021). Alternatively, hay may delay the latency to start cross-sucking behavior, which can become a habit for life (Keil et al., 2001). Therefore, the objective of this study was to assess whether providing pelleted timothy hay to pair-housed dairy calves could affect the duration, frequency, and latency in days to start cross-sucking behavior surrounding the daily milk feedings for the first 21 d after pairing. We hypothesized that offering hay would reduce the daily duration and frequency of cross-sucking bouts, while increasing the latency in days to being cross-sucking behavior.

This randomized control trial was conducted at the Penn State Dairy Research Farm (IACUC: PROTO202302536) as part of a concurrent study investigating the effects of pelleted hay on paired calf growth performance and diarrhea outcomes. A subset of paired calves (n = 12 pairs; 6 pairs negative control, **CON**; 6 pairs timothy hay pellets, **Hay**) born between February 2024 and March 2024 were enrolled at pairing (5 ± 3 d of age; mean ± SD). Salter et al. (2021) observed calves cross-sucking 1.67% of the time during their sampling period with a definition of cross-sucking directed at the head, belly, or tail; our definition for cross-sucking differed and was limited to calves cross-suckling a calf's underside where producers might observe udder damage in mature cattle (Keil et al., 2001; Table 1). We calculated this to the percentage spent cross-sucking in hours per day. We estimated that calves would cross-suck 0.10 h/d with a variability of 0.25 min. We expected Hay to cut the cross-sucking duration in half; therefore, we required 6 pairs per treatment at a power of 80% and an α of 0.05.

**Table 1:** Ethogram describing behavior observed in pair-housed calves (n = 12 pairs) enrolled in a randomized control trial investigating the effects of pelleted timothy hay on the daily cross-sucking duration, latency, and frequency surrounding milk feeding (6 h/d) for the first 21 d after pairing

Behavior	Description
Cross-sucking	One calf suckling on the navel, udder area, or between the back legs of the other calf
Bout	A single bout began if a cross-sucker calf contacted the defined areas of the receiving calf and ended when the cross-sucker broke contact with the defined areas of the receiving calf for ≥10 s
Frequency of bouts	The sum of all cross-sucking bouts within a pair in an observation period
Duration in seconds	The sum of all contact time for each bout of cross-sucking within a pair in an observation period
Latency in days	The time in days until at least one cross-sucking bout occurred within a pair

Video cameras (Amcrest UltraHD 5 MP Outdoor POE Camera, Amcrest Industries LLC, Houston, TX) were placed at an angle above and behind a row of calf pens on one side of the barn so that calves were visible at all times and recorded calf behavior continuously surrounding milk feedings (6 h/d; 1 h before milking feeding and 2 h after milk feeding twice daily). Treatment assignments were balanced in blocks of 6 for a concurrent study, and due to the technological limitations, we enrolled one block where each pair was housed in a pen under a camera. Video footage was stored using Synology software (Synology RS1221RP, Synology Inc., Taipei, Taiwan) and then downloaded onto Seagate hard drives (Seagate Technology Holdings plc, Fremont, CA) for storage until observer coding.

All calves were fed 7.4 L/d (135 g/L) of Land O'Lakes Amplifier Max (Land O'Lakes Inc., Arden Hills, MN) milk replacer split into 2 daily feedings by bottle from birth. A texturized calf starter grain (East Gate Feed and Grain, Reedsville, PA) was available ad libitum to all calves by 2 buckets and 2 Braden bottles (Coburn Co., Whitewater, WI) at the front of the pen after pairing. Water was provided ad libitum by bucket from birth. Pairs that were assigned to receive pelleted timothy hay (Hay) were offered a pelleted timothy hay (Mountain Sunrise Feed, Beryl, UT) in a trough (95.85 cm long × 15.24 cm wide × 10.16 cm high) placed at the inside back of the pen at pairing. Once a week, a sample of timothy hay pellets and calf starter grain was taken before feeding. The samples were frozen at −20℃. Later, each sample was weighed, dried by a forced-air oven (VWR Scientific 1690 HAFO Series Oven, Radnor, PA) at 55℃ for 48 h, and weighed again to calculate the % DM. After drying, monthly composite samples were made using a Wiley mill (Thomas Scientific, Swedesboro, NJ). Samples were sent to Cumberland Valley Analytical Services (Waynesboro, PA) for nutrient analysis including sugar, NDF, ADF, % CP, % fat, and ash. Calf starter was also evaluated for % starch. Feed analysis is available from the corresponding author.

Cross-sucking behavior was coded using BORIS (https://www.boris.unito.it/) by 2 trained observers after achieving an interobserver agreement of ĸ ≥ 0.80. To calculate agreement, observers coded cross-sucking for 360 min of video footage on 2 different days for 2 different pairs of calves. The calculation included agreement for cross-sucking bouts, and a separate calculation was performed for cross-sucking duration using BORIS software. One hour before each daily milk meal and the 2 h after each daily milk meal were recorded and observed for cross-sucking. An ethogram of each behavior is available in Table 1.

All statistical analyses were performed using SAS (version 9.4, SAS Institute Inc., Cary, NC). We declared significance at *P*  ≤ 0.05. Data were imported from BORIS into Excel (Microsoft Office Excel version 16.78, Microsoft Corp.) and checked for completeness. Data were verified for normality using the Shapiro–Wilk test, and probability distribution plots. Data were non-normally distributed (Shapiro–Wilk criteria <0.95) and were common log-transformed with a 1.0 correction factor for modeling. The homogeneity of variance was assessed using the Levene test and confirmed for the transformed data. Transformed data were then confirmed by visually examining the residuals from the linear mixed models and plotting the predicted residuals against the 95% CI. Any suspect outliers outside of the 95% CI from the predicted residuals were tested for model leverage using Cook's distance. A Cook's distance of ≥1 SD from the other predicted LSM residuals was considered a candidate for removal, but none were observed. We selected our covariance structures for best model fit using the lowest Akaike information criterion for Toeplitz, compound symmetry, and first-order autoregressive structures. Compound symmetry had the best model fit for all outcomes. The experimental unit was pair.

A mixed linear regression model was used to assess the effect of Hay, day, and the Hay × day interaction with the frequency of cross-sucking bouts and the daily duration of cross-sucking. The Hay × day interaction, Hay and day were fixed effects. We expected an interaction of Hay with day for cross-sucking behavior because we expected the calves to increase their DMI of solid feed as they increased with age, and rumination may have a protective effect against non-nutritive oral behavior as observed by others (Wang et al., 2022). We used day as a repeated measure with pair as the subject. The back-transformed geometric means and geometric 95% CI are reported. Pearson's chi-squared test was used to assess the association of Hay with the latency in days to begin cross-sucking.

We found no association of Hay with the daily duration of cross-sucking bouts surrounding milk feeding and no Hay × day interaction (*P* > 0.05). The geometric mean duration of cross-sucking surrounding milk feeding for CON was 54.7 (95% CI: 49.3 to 60.5 s) and for Hay was 59.4 (95% CI: 53.3 to 65.5 s). The cross-sucking bout daily duration in minutes within a pair across all observations is in Figure 1a. The variability of the average daily duration of cross-sucking bouts throughout the study is in Figure 1b. As shown in Figure 1, cross-sucking reflects individual characteristics, one pair spent an extreme amount of 20 min engaging in cross-sucking, but most behavior occurred in under a minute per day, with the average of each bout being represented in seconds. Note that while we had extreme outliers before the data were transformed for cross-sucking duration, the median for the daily duration of cross-sucking bouts was 0.19 min for CON and 0.28 min for Hay pairs. There was no association of Hay with the latency in days to begin cross-sucking behavior (*P* > 0.05). The latency for CON pairs to begin cross-sucking was 0 d (95% CI: 0.00 to 0.00 d) while the latency for Hay pairs to begin cross-sucking was 1.67 (95% CI: 0.81 to 2.52) days. We also found no association of Hay with the frequency of daily cross-sucking bouts surrounding milk feeding and no Hay × day interaction, and the frequency by pair is in Figure 2a and by calf is in Figure 2b (*P* > 0.05). The geometric mean frequency of cross-sucking bouts surrounding milk feeding for CON was 7.8 (95% CI 7.5 to 8.1 bouts) and for Hay was 8.1 (95% CI: 7.7 to 8.4 bouts). All calves enrolled in the trial performed cross-sucking at some point during the study. The average hay DMI throughout the study was negligible (Hay 0.11 ± 0.06 kg/d; mean ± SD), reaching 0.22 kg/d on d 21. Calves consumed negligible grain throughout the study, though intakes were similar (Hay 0.26 ± 0.01 kg/d vs. CON 0.25 ± 0.01 kg/d; *P* > 0.05).

**Figure 1:**
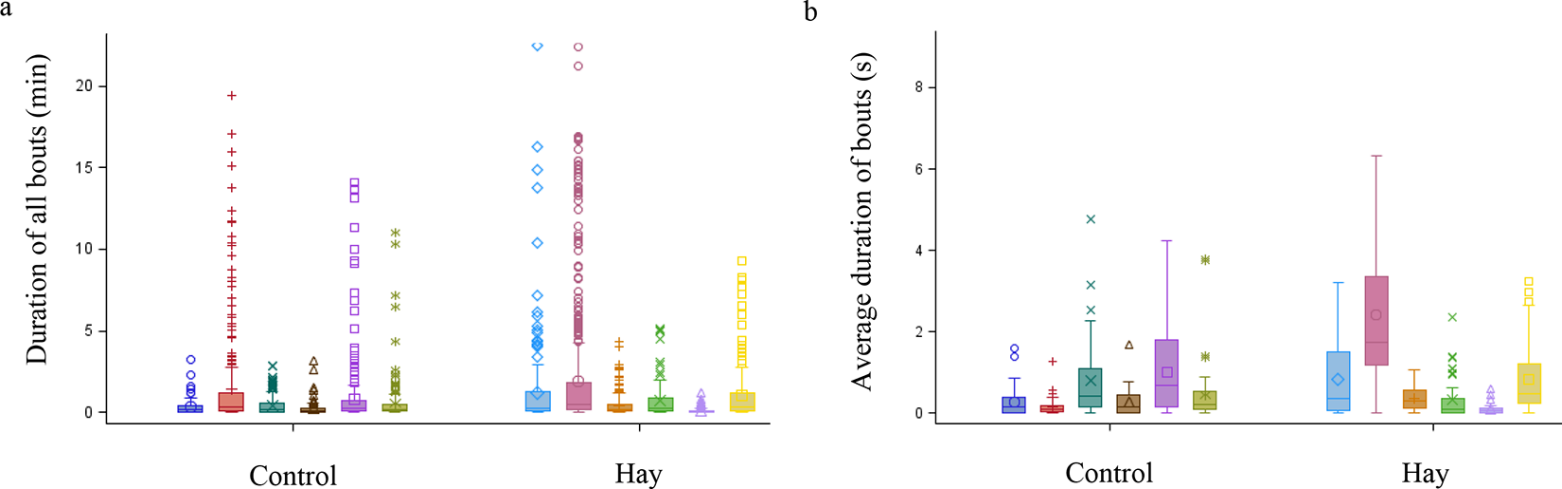
A box and whisker plot demonstrating the minimum (bottom whisker), first quartile (bottom box line), median (center line), mean (symbol), third quartile (top box line), maximum (1.5 times the interquartile range, top whisker), and outliers for the duration in minutes of each cross-sucking bout that occurred by pair (n = 1,213 observations for control and 1,378 for hay; panel a), and the average duration of each cross-sucking bout by pair throughout the study (panel b). Calves were observed daily for the 6 h surrounding milk feeding (n = 12 pairs) fed either pelleted timothy hay (Hay) or not for 21 d (control; panel a; *P* > 0.05).

**Figure 2:**
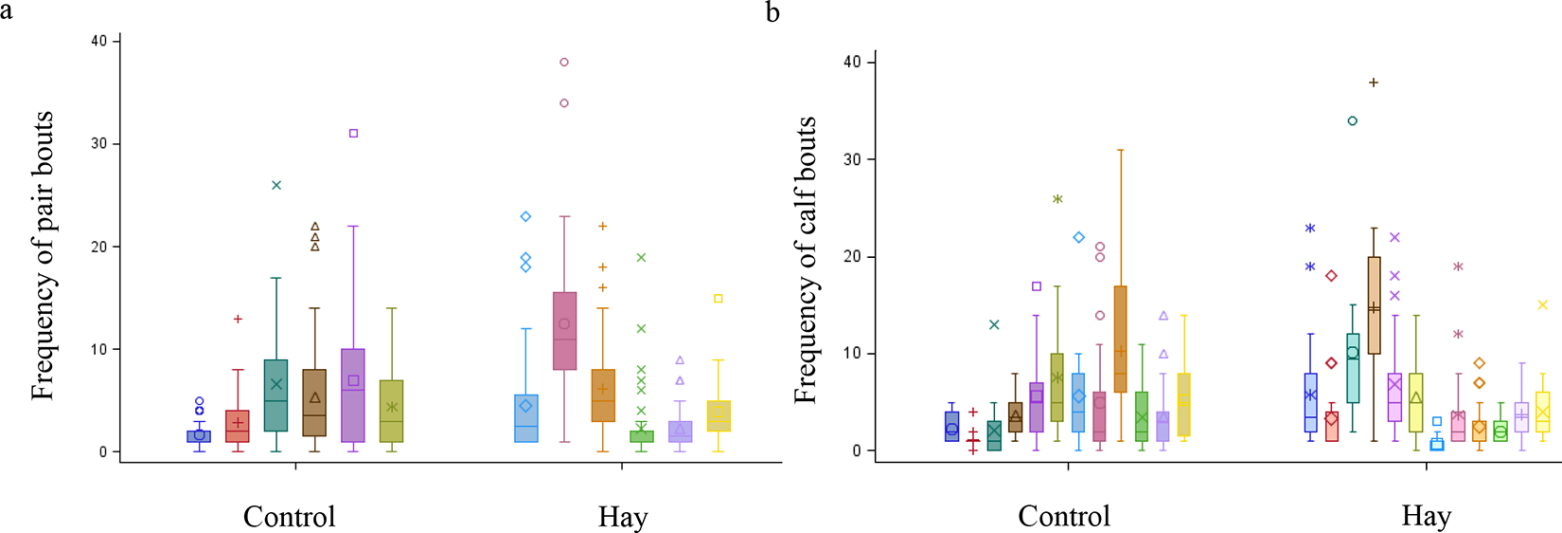
A box and whisker plot demonstrating the minimum (bottom whisker), first quartile (bottom box line), median (center line), mean (symbol), third quartile (top box line), maximum (1.5 times the interquartile range, top whisker), and outliers for the frequency of each cross-sucking bout by pair (panel a), and frequency of cross-sucking bouts by calf (panel b) throughout the 21 d. Calves were observed daily for the 6 h surrounding milk feeding (n = 12 pairs) fed either pelleted timothy hay (Hay) or not for 21 d. There was no association of hay with cross-sucking bout frequency (*P* > 0.05).

Our findings in this study directly contrast with our hypotheses, as we anticipated that offering Hay to calves would reduce their cross-sucking duration and bouts by providing an alternative activity. Cross-sucking was often infrequent at a minute surrounding milk feeding. It is possible that cross-sucking was infrequent in our study because we offered calves other items that discourage cross-sucking behavior such as Braden bottles (Salter et al., 2021), teat nipples, and feeding more than 7 L/d of milk (as reviewed by Plaugher and Cantor, 2025). However, despite the behavior occurring for a low amount of time, all calves cross-sucked, and cross-sucking happened immediately after pairing. Thus, we suggest that in this context, pelleted hay may not discourage cross-sucking in calves, but perhaps it helps with cross-sucking in more barren environments. Alternatively, our sampling strategy (6 h around milk feeding) is different than others such as Zhang et al. (2021) who scan sampled every 5 min over daylight hours, or Ude et al. (2011) who observed for 20 min after milk feeding, and so on. For example, Salter et al. (2021) found that only 30% of the cross-sucking behavior observed was related to our definition of suckling the teats and underside of the calf. Perhaps forage does not discourage cross-sucking behavior, often referred to as intersucking, directed at the belly or udder area but does discourage other types of non-nutritive oral behavior such as suckling the ears (Roth et al., 2008). We suggest that more work is needed to identify why calves cross-suck, and motivations to engage in the behavior surrounding pairing.

We hypothesized that a timothy hay pellet would reduce the frequency and duration of cross-sucking bouts, yet we observed no relationship. Our work disagrees with the literature, but we want to highlight here and elsewhere that direct comparisons of cross-sucking behavior among studies are impossible due to differences in definitions, observation periods, and grouping strategies of calves (Roth et al., 2008; Ude et al., 2011; Velasquez-Munoz et al., 2023). Thus, all work presented is interpreted with an abundance of caution because only one study has looked at strategies to mitigate cross-sucking behavior in paired calves managed like those in our study (Salter et al., 2021). For example, Ude et al. (2011) found that providing calves from 25 to 51 d of age with a post-milk meal enrichment with hay reduced the frequency of cross-sucking bouts, but it did not affect the duration. Zhang et al. (2021) observed that scented strawberry hay discouraged calf cross-sucking compared with brushes and chains. Roth et al. (2008) observed that as time eating forage increased, cross-sucking decreased in calves. A factor that may have affected our study outcomes was that our calves were pair-housed rather than housed in larger groups (Roth et al., 2008; Ude et al., 2011; Zhang et al., 2021). It has been suggested that cross-sucking is a multifaceted behavior that is affected by both nutritional and social factors, as pairs of calves that mutually suck each other have been observed into the postweaning period (Endres and Schwartzkopf-Genswein, 2018). Plus, cross-sucking is a lifetime habit (Keil et al., 2001). An investigation into the social network of calves found that in group settings calves generally associate with the same calves and that familiarity grows stronger as time progresses (Vázquez-Diosdado et al., 2023). Knowing this, we suggest that pair-housed calves may form stronger bonds and relationships than group-housed calves, which could fuel cross-sucking behavior in pairs that exhibit it. This is exhibited in our pairs, where some calves expressed cross-sucking bouts at a highly variable frequency throughout the study, whereas other pairs cross-suckled rarely. However, we also must mention that in calves offered 7.4 L/d milk, cross-sucking was often infrequent, but persistent. We suggest that future research needs to identify how cross-sucking develops, and how much time spent engaging in the behavior increases the risk of poor udder health (Mahmoud et al., 2016).

Latency to begin cross-sucking has never been investigated, and our study found no association of feeding timothy hay with the latency to start the behavior. It is known that milk allowance directly affects cross-sucking behavior (Jensen, 2003) and may have some effect on latency to begin cross-sucking. However, it is possible that had we offered forage at birth, calves may have interacted with it differently regarding latency to start cross-sucking after pairing. It is important to note that the DMI of the hay pelleted used in this study was low in our calves at 0.1 kg/d. Young calves do not begin consuming large amounts of solid feed until 2 to 3 wk of age (Drackley, 2008), and calves in our trial may not have consumed enough pelleted hay. We watched the first 21 d because we were interested in latency to develop the behavior, and because others have observed that the younger a calf ruminates, the less likely the calf is to perform non-nutritive oral behavior (Wang et al., 2022). Thus, we expected calves to be drawn to the pelleted hay in their environment. Perhaps pelleted hay does not offer the same benefits to calves as long-stem hay. Long-stem hay has discouraged cross-sucking in calves because of increased feeding times, gut fill, and mastication time, which provides a buffer in the rumen (Ude et al., 2011; Horvath and Miller-Cushon, 2019; Zhang et al., 2021). Perhaps the pelleted nature of the hay decreased the time spent in the rumen, thus not offering the same benefits to calves as other hay varieties, though this warrants investigation.

In conclusion, pelleted timothy hay did not affect the daily duration and frequency of cross-sucking behavior in pair-housed calves surrounding milk feeding (e.g., 6 h/d) for the first 21 d. Offering hay to paired calves also did not affect the latency to begin cross-sucking behavior, though cross-sucking occurred immediately after pairing (e.g., 0 to 1 d). Although we did not find a treatment effect, we did observe a very low frequency of cross-sucking (e.g., 8 bouts per observation period) and a low duration (e.g., less than a minute per observation period) surrounding milk feeding in our paired calves. We suggest that this is likely because we used Braden bottles as an additional outlet for cross-sucking behavior and fed calves at least 7.4 L/d milk replacer by nipple bottle. We recommend that dairy farmers pair-housing calves use Braden bottles and feed increased planes of milk to minimize the amount of cross-sucking that occurs. We suggest future research should further investigate why paired calves engage in cross-sucking behavior immediately after pairing.
